# Quantitative profiling of housekeeping and Epstein-Barr virus gene transcription in Burkitt lymphoma cell lines using an oligonucleotide microarray

**DOI:** 10.1186/1743-422X-3-43

**Published:** 2006-06-06

**Authors:** Michele Bernasconi, Christoph Berger, Jürg A Sigrist, Athos Bonanomi, Jens Sobek, Felix K Niggli, David Nadal

**Affiliations:** 1 Division of Infectious Diseases and Division of Oncology, University Children's Hospital of Zurich, August Forel-Strasse 1, CH-8008 Zurich, Switzerland; 2 Functional Genomics Center of the University of Zurich, Winterthurerstrasse 190CH-8057 Zurich, Switzerland

## Abstract

**Background:**

The Epstein-Barr virus (EBV) is associated with lymphoid malignancies, including Burkitt's lymphoma (BL), and can transform human B cells in vitro. EBV-harboring cell lines are widely used to investigate lymphocyte transformation and oncogenesis. Qualitative EBV gene expression has been extensively described, but knowledge of quantitative transcription is lacking. We hypothesized that transcription levels of *EBNA1*, the gene essential for EBV persistence within an infected cell, are similar in BL cell lines.

**Results:**

To compare quantitative gene transcription in the BL cell lines Namalwa, Raji, Akata, Jijoye, and P3HR1, we developed an oligonucleotide microarray chip, including 17 housekeeping genes, six latent EBV genes (*EBNA1*, *EBNA2*, *EBNA3A*, *EBNA3C*, *LMP1*, *LMP2*), and four lytic EBV genes (*BZLF1*, *BXLF2*, *BKRF2*, *BZLF2*), and used the cell line B95.8 as a reference for EBV gene transcription. Quantitative polymerase chain reaction assays were used to validate microarray results. We found that transcription levels of housekeeping genes differed considerably among BL cell lines. Using a selection of housekeeping genes with similar quantitative transcription in the tested cell lines to normalize EBV gene transcription data, we showed that transcription levels of *EBNA1 *were quite similar in very different BL cell lines, in contrast to transcription levels of other EBV genes. As demonstrated with Akata cells, the chip allowed us to accurately measure EBV gene transcription changes triggered by treatment interventions.

**Conclusion:**

Our results suggest uniform *EBNA1 *transcription levels in BL and that microarray profiling can reveal novel insights on quantitative EBV gene transcription and its impact on lymphocyte biology.

## Background

The B-cell-tropic Epstein-Barr virus (EBV) is associated with lymphoid malignancies, including Burkitt's lymphoma (BL), Hodgkin's disease, and post-transplant lymphoproliferative disease [1]. Consistent with its role as a tumor virus, EBV can transform human B cells in vitro [2], and EBV-harboring cell lines constitute a key research tool to study pathogenic events leading to lymphocyte transformation and oncogenesis.

As noted in studies of tumors and cell lines, expression of latent EBV genes contributes to cell transformation, and these studies have resulted in the description of three EBV latency programs [3,4]. The latency I program expresses the EBV nuclear antigen (EBNA) 1 gene and is characteristic of BL. The latency II program expresses EBNA1 plus the latent membrane proteins (LMP) 1 and LMP2 and is seen in Hodgkin's disease and the epithelial malignancy nasopharyngeal carcinoma. The latency III program involves expression of all six EBNAs, LMP1, 2A, and 2B, and EBV-encoded RNAs. It is found in EBV-driven lymphoproliferations of the immunocompromised host and in EBV-transformed lymphoblastoid cell lines (LCLs).

Recently, an EBV gene expression program that closely matches the EBV growth-promoting latency III program was reported in a subset of BL [5]. Notably, latent EBV infection can be disrupted by expression of the master regulator lytic EBV gene *BZLF1 *that initiates EBV replication, ultimately resulting in the assembly of new EBV particles and their release upon cell lysis [6,7]. This observation ignited great interest in a possible new therapeutic strategy against EBV-harboring tumors: inducing lytic EBV infection with subsequent cell lysis [8].

Quantitative characterization of EBV gene transcription would allow a more in-depth analysis of the patterns and dynamics of EBV gene transcription in different cellular backgrounds that, in turn, could reveal important regulatory mechanisms governing the maintenance of EBV latent infection, host cell transformation, and reactivation of lytic infection. Thus, a research tool to quantify simultaneous EBV gene transcription is desirable. Unfortunately, although EBV gene expression in EBV-infected cell lines has been studied extensively, only non- or semi-quantitative methods, such as Northern blotting or Southern reverse-transcription polymerase chain reaction (PCR) assays, have been used [4] and, little is known about the quantitative EBV gene transcription levels in infected B-cells.

We hypothesized that the transcription levels of *EBNA1*, the EBV gene essential for EBV persistence in the infected cell, are similar in rather different BL cell lines. To test this hypothesis, we developed an EBV oligonucleotide (ODN) microarray chip applicable to different cellular backgrounds and used it to perform comparative quantification of latent and lytic EBV gene transcription normalized to housekeeping genes in a limited set of EBV-harboring BL cell lines.

## Results

### Selecting housekeeping genes to normalize EBV gene transcription in BL cell lines

The first step in quantifying gene transcription is to identify genes that can be used as controls. Internal control genes, often referred to as housekeeping genes, should not vary among the tissues or cells under investigation. Unfortunately, considerable variability has been reported in the transcription of many housekeeping genes [9,10].

To build our microarray chip, we started with housekeeping genes derived from two groups of the Human Gene Expression Index (HuGE) [9] and for which probes were already described in the Church set of human probes [11]. We began with those with either the highest transcription levels (e.g., *RPL37A*, *KIAA0220*, *CLU*, *MT2A*, *FTL*) or the most constant transcription (e.g., *PSMD2*, *PSMB3*, *TCFL1*, *H3F3A*, *PTDSS1*, *KARS*, *AAMP*, *384D8-2*). In addition, we included commonly used housekeeping genes (*ACTB*, *c-yes*, *MHCL*, *HMBS*) [12] (Table 1A).

**Table 1A:** Probes for housekeeping genes

Oligo Name	Unigene	nt	Transcript Length (nt)	Distance from 3' (nt)	Tm (°C)	GC (%)	Design	Sense Probe Sequence (5' to 3')
RPL37A	Hs.433701	70	1059	607	78.3	50.0	CS	AGGCCTTCCCGAGAAAGTGCTTAGCCTTGTTGATGATCCAAGGAACCACATAGAGAACCAAGACGAGTGC
KIAA0220	Hs.110613	70	1121	416	82.4	60.0	CS	TGGAACCATCATCACCCGAACCCAAGAGGCGGAGGGTCGGTGACGTGGAACCGTCACGCAAACCCAAGAG
CLU	Hs.75106	70	1676	903	82.4	60.0	CS	TTTCCCAAGTCCCGCATCGTCCGCAGCTTGATGCCCTTCTCTCCGTACGAGCCCCTGAACTTCCACGCCA
MTA2	Hs.118786	70	1929	154	78.8	51.4	CS	GCAAGAAGTTACGACACGTACACAACGACAGAACAACAGAGAAGACCCCGAAGACCACTAGCACGACCGT
384D8-2.2	Hs.356523	71	2281	334	80.6	52.2	CS	CGAAGGAAAGTGGAGCTCTTCATCGCCACCTCCCAGAAGTTTATCCAGGAGACAGAGCTGAGCCAGCGCA

FTL	Hs.118786	70	1929	154	78.8	51.4	CS	CTCTCTCTTTCAGGCCTCAACAGGCACTGTATTCATTGCCAATGTTCCAAATTATCAAATTCAAGTGAAT
PSMD2	Hs.74619	70	2828	122	75.9	44.3	CS	TATCTTCGGAAGAACCCCAATTATGATCTCTAAGTGACCACCAGGGGCTCTGAACTGTAGCTGATGTTAT
PSMB3	Hs.82793	70	692	42	79.4	52.8	CS	ATCATCGAGAAGGACAAAATCACCACCAGGACACTGAAGGCCCGAATGGACTAACCCTGTTCCCAGAGCC
T CFL1	Hs.2430	70	1324	153	76.5	46.2	CS	CCCCGAGCCTTGCGCCAGAAAATTGTCATTAAATGAAGAGATGTCTAGTCCTCAGAAACTTCTTTCCTGC
H3F3A	Hs.181307	70	1305	20	73.6	38.5	CS	GAGTTGTCCTACATGCAAGTACATGTTTTTAATGTTGTCTGTCTTCTGTGCTGTTCCTGTAAGTTTGCTA
PTDSS1	Hs.77329	70	2504	242	77.7	48.6	CS	GTAGCTGCCTGCATAGGAGCCTCGCTTCCGATTATTCCCTTCCCAATATTATTCATCCAGACTTAGCCAC
KARS	Hs.3100	70	1997	142	73.6	38.6	CS	GCAACCACTGATACACTGGAAAGCACAACAGTTGGCACTTCTGTCTAGAAAATAATAATTGCAAGTTGTA
AAMP	Hs.83347	70	1762	622	81.2	57.1	CS	ACCTTGGCCATCTATGACCTGGCTACGCAGACTCTTAGGCATCAGTGTCAGCACCAGTCGGGCATCGTGC

β-actinsense	Hs.288061	68	1841	431	92.7	50.0	PE	TTAAAAACTGGAACGGTGAAGGTGACAGCAGTCGGTTGGAGCGAGCATCCCCCAAAGTTCACAATGTG
β-actin.70 mer	Hs.288061	71	1841	700	96.0	56.3	PE	CCTGGCACCCAGCACAATGAAGATCAAGATCATTGCTCCTCCTGAGCGCAAGTACTCCGTGTGGATCGGCG
c-yes.70 mer	Hs.194148	70	4343	1249	96.7	62.9	PE	CTCGGCTCACTGCAAGCTCTGCCTCCCAGGTTCACACCATTCTCCTGCCTCAGCCTCCCGAGTAGCTGGG
c-yes.2	Hs.194148	70		539	73.6	52.2	CS	CATGCAAGTTGGCAGTGGTTCTGGTACTAAAAATTGTGGTTGTTTTTTCTGTTTACGTAACCTGCTTAGT
MHCI.70 mer	Hs.379218	70	2290	600	92.7	55.7	PE	CTCAGATAGAAAAGGAGGGAGCTACTCTCAGGCTGCAAGCGGCAACAGTGCCCAGGGCTCTGATGTGTCT
HMBS.70 mer	Hs.82609	70	1536	1300	97.7	57.0	PE	ACGGCAATGCGGCTGCAACGGCGGAAGAAAACAGCCCAAAGATGAGAGTGATTCGCGTGGGTACCCGCAA
HMBS.2	Hs.82609	70		164	82.4	52.2	CS	TGCTGTCCAGTGCCTACATCCCGGGCCTCAGTGCCCCATTCTCACTGCTATCTGGGGAGTGATTACCCCG
EF1.70 mer	Hs.181165	71	1833	500	91.8	50.7	PE	GGCAAGCCCATGTGTGTTGAGAGCTTCTCAGACTATCCACCTTTGGGTCGCTTTGCTGTTCGTGATATGAG

CS: Church set; PE: Primer Express

Next we determined the suitability of the genes for our assay. The marmoset cell line B95.8 was selected as the reference line because it expresses all of the latent genes and most of the lytic EBV genes under normal culture conditions [13]. B95.8 is of primate origin, and we focused particularly on probes derived from human housekeeping gene sequences that would hybridize with the same efficiency to B95.8 gene sequences. RNAs from human BL cell lines (e.g., BJAB, Namalwa, Raji, Akata, Jijoye, and P3HR1) were used in self-vs-self hybridizations. Transcription levels for 13 of 17 housekeeping genes were detected over background in these cell lines, and 12 housekeeping genes showed levels similar to those found in B95.8 cells (Table 2). The coefficient of variation (CV), calculated as the ratio of the standard deviation (SD) to the mean of the transcription detected in all cell lines tested, ranged from 0.17 for *ACTB *to 2.24 for *CLU*. Probes with a CV > 0.5 were eliminated. Probes with a mean transcription signal > two SD and significant transcription in B95.8 were selected for the normalization housekeeping gene set. Using these criteria, we identified eight housekeeping genes *PSMD2*, *PSMB3*, *TCLF1*, *PTDSS1*, *AAMP*, *ACTB*, *c-yes*, and *HMBS *for the normalization housekeeping gene set (Table 2).

**Table 2:** Expression profiles of 17 housekeeping genes in a panel of cell lines

	B95.8			BJAB			Namalwa			Raji			Akata			Jijoye			P3HR1			All cell lines		
High expression	Mean	SD	CV	Mean	SD	CV	Mean	SD	CV	Mean	SD	CV	Mean	SD	CV	Mean	SD	CV	Mean	SD	CV	Mean	SD	CV

*RPL37A*	0.36	0.05	0.15	0.33	0.02	0.06	1.31	0.18	0.14	0.27	0.21	0.76	1.69	0.02	0.01	2.05	0.01	0.01	1.35	0.05	0.04	1.10	0.68	0.62
*KIAA0220*	0.06	0.01	0.12	2.33	0.60	0.26	1.56	0.07	0.05	2.98	0.79	0.27	1.64	0.03	0.02	2.02	0.01	0.01	1.37	0.02	0.01	1.63	0.87	0.54
*CLU*	1.62	0.02	0.01	0.21	0.04	0.21	0.06	0.03	0.41	n.d.		0.00	0.13	0.04	0.33	0.20	0.06	0.28	0.20	0.02	0.12	0.26	0.59	2.24
*MTA2*	0.00	0.01	12.49	0.02	0.02	1.13	n.d.		0.00	n.d.		0.00	n.d.		0.00	n.d.		0.00	n.d.		0.00			
*384D8-2.2*	0.27	0.05	0.17	0.32	0.04	0.13	0.35	0.04	0.11	n.d.		0.00	0.36	0.06	0.16	0.70	0.21	0.30	0.64	0.04	0.07	0.29	0.43	1.47

Constant expression	Mean	SD	CV	Mean	SD	CV	Mean	SD	CV	Mean	SD	CV	Mean	SD	CV	Mean	SD	CV	Mean	SD	CV	Mean	SD	CV

*FTL*	1.49	0.08	0.05	0.20	0.01	0.05	0.10	0.05	0.54	n.d.		0.00	0.33	0.12	0.36	0.51	0.25	0.49	0.75	0.10	0.13	0.45	0.59	1.31
***PSMD2***	**0.89**	**0.04**	**0.04**	**2.38**	**0.67**	**0.28**	**1.08**	**0.05**	**0.04**	**0.24**	**0.01**	**0.06**	**1.67**	**0.02**	**0.01**	**1.46**	**0.20**	**0.14**	**1.04**	**0.15**	**0.14**	**1.22**	**0.63**	**0.52**
***PSMB3***	**1.49**	**0.02**	**0.01**	**2.35**	**0.62**	**0.27**	**1.61**	**0.12**	**0.08**	**0.62**	**0.22**	**0.36**	**1.70**	**0.00**	**0.00**	**2.03**	**0.00**	**0.00**	**1.35**	**0.04**	**0.03**	**1.57**	**0.51**	**0.33**
***TCLF1***	**0.53**	**0.02**	**0.03**	**0.72**	**0.01**	**0.02**	**1.43**	**0.13**	**0.09**	**0.20**	**0.06**	**0.30**	**0.51**	**0.21**	**0.42**	**1.26**	**0.32**	**0.25**	**1.18**	**0.11**	**0.09**	**0.90**	**0.40**	**0.52**
*H3F3A*	1.71	0.03	0.02	2.44	0.76	0.31	1.64	0.13	0.08	7.38	4.68	0.63	1.69	0.00	0.00	2.08	0.01	0.01	1.35	0.04	0.03	2.44	2.03	0.83
***PTDSS1***	**1.40**	**0.00**	**0.00**	**2.45**	**0.76**	**0.31**	**1.51**	**0.07**	**0.04**	**4.87**	**1.42**	**0.29**	**0.95**	**0.02**	**0.02**	**1.92**	**0.09**	**0.05**	**1.35**	**0.03**	**0.02**	**1.98**	**1.25**	**0.53**
*KARS*	1.19	0.03	0.03	2.45	0.77	0.31	1.62	0.12	0.07	6.40	3.35	0.52	1.66	0.02	0.01	2.08	0.01	0.00	1.35	0.05	0.03	2.22	1.75	0.79
***AAMP***	**0.57**	**0.01**	**0.02**	**0.62**	**0.01**	**0.01**	**0.59**	**0.04**	**0.07**	**0.13**	**0.04**	**0.31**	**0.55**	**0.07**	**0.12**	**1.00**	**0.18**	**0.18**	**1.29**	**0.02**	**0.01**	**0.71**	**0.35**	**0.50**

Common	Mean	SD	CV	Mean	SD	CV	Mean	SD	CV	Mean	SD	CV	Mean	SD	CV	Mean	SD	CV	Mean	SD	CV	Mean	SD	CV

***βactin.sense***(*ACTB*)	**1.73**	**0.04**	**0.02**	**1.68**	**0.28**	**0.17**	**1.21**	**0.07**	**0.06**	**2.28**	**0.23**	**0.10**	**0.78**	**0.13**	**0.16**	**1.72**	**0.24**	**0.14**	**1.34**	**0.01**	**0.00**	**1.49**	**0.46**	**0.31**
***βactin.70 mer***(*ACTB*)	**1.69**	**0.02**	**0.01**	**1.00**	**0.24**	**0.24**	**1.42**	**0.04**	**0.02**	**1.16**	**0.05**	**0.05**	**1.65**	**0.01**	**0.01**	**1.44**	**0.01**	**0.01**	**1.33**	**0.05**	**0.04**	**1.37**	**0.23**	**0.17**
***c-yes.70 mer***	**1.32**	**0.02**	**0.02**	**2.05**	**0.42**	**0.20**	**1.51**	**0.12**	**0.08**	**0.92**	**0.12**	**0.13**	**1.70**	**0.02**	**0.01**	**2.05**	**0.00**	**0.00**	**1.35**	**0.06**	**0.04**	**1.67**	**0.61**	**0.37**
*c-yes.2*	*0.45*	*0.05*	*0.10*	*0.11*	*0.03*	*0.32*	*n.d.*		*0.00*	*n.d.*		*0.00*	*0.05*	*0.01*	*0.28*	*0.13*	*0.01*	*0.11*	*0.08*	*0.01*	*0.11*			
*MHCL.70 mer*	*1.73*	*0.05*	*0.03*	*0.48*	*0.13*	*0.27*	*1.57*	*0.07*	*0.05*	*0.80*	*0.01*	*0.01*	*1.69*	*0.02*	*0.01*	*0.04*	*0.04*	*1.18*	*1.27*	*0.02*	*0.02*	*1.10*	*0.62*	*0.55*
***HMBS.2***	**1.03**	**0.02**	**0.13**	**2.42**	**0.72**	**0.30**	**1.59**	**0.10**	**0.06**	**2.57**	**0.39**	**0.15**	**1.47**	**0.03**	**0.02**	**1.97**	**0.05**	**0.03**	**1.35**	**0.05**	**0.04**	**1.52**	**0.33**	**0.22**

CV: coefficient of variation; n.d.: not detected.; SD: Standard deviation; bold letters indicate gene-sets that were considered to be constantly detectable across cell lines, and were selected to be used as normalization-set in further experiments. Probe sets that showed a CV < 0.6 were selected for the normalization housekeeping gene set.

### Selection of EBV-specific probes

During latency, EBV expresses a limited set of the 85 predicted open reading frames from its genome [14]. Seven EBNAs, three LMPs, and the non-coding EBERs can be expressed in B-cells. Reactivation of EBV occurs via expression of its immediate early lytic gene *BZLF1 *and a subsequent cascade of gene activation [15].

**Figure 1:**
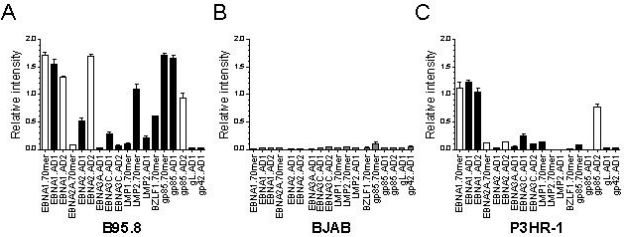
**Selection of microarray probes**. The specificity of EBV gene probes was tested in the reference cell line B95.8 as positive control (A) and in the EBV-negative cell line BJAB (B). The P3HR1 strain of EBV is characterized by a large deletion in the region coding for *EBNA2 *and was used to validate the specificity of *EBNA2 *probes (C). Black bars represent probes considered specific and selected for the final version of the chip. Mean ± SEM values (with background subtracted) were normalized to the set of eight housekeeping genes. Robust signals were measured for most latent and lytic EBV genes in the reference cell line B95.8. White bars indicate probes that were not selected.

A microarray targeting specific viral genes depends heavily on selection strategy for the probe design. To accurately monitor EBV transcription, we designed probes specific for selected EBV genes: some from the latent phase (e.g., *EBNA1*, *EBNA2*, *EBNA3A*, *EBNA3C*, *LMP1*, and *LMP2*) and some from lytic EBV infection (e.g., *BZLF1/ *Zta, *BXLF2/*gp85, *BKRF2/*gL and *BZLF2/*gp42) (Table 1B). We used B95.8 RNA as positive control and RNA from the EBV-negative BL cell line BJAB as a negative control to eliminate probes that cross-hybridize with cellular genes (Fig. 1). RNAs from B95.8 and BJAB cells were compared in self-vs-self experiments. EBV probes giving a signal with B95.8 RNA but not with BJAB RNA were selected. We tested three probes for *EBNA1*, *EBNA2*, and *BXLF2*/gp85 and two probes for *LMP2 *designed with either PE or AD probe design software. All probes for *EBNA1 *had good sensitivity and specificity, and the probes for *EBNA2 *and *BXLF2*/gp85 designed with PE showed a nonspecific signal when hybridized to BJAB, as well as one of the *EBNA2 *probes designed with AD (EBNA2_AD2). To sample the efficiency of the chip design, we also used the cell line P3HR1, which has a deletion in the *EBNA2 *gene [16]. No signal over background was detected with the *EBNA2 *probe (EBNA2_AD1) (Fig. 1C). These results indicate that dedicated microarray design software and primer design software can select for sensitive and specific probes but not with 100% accuracy.

**Table 1B:** Probes for Epstein-Barr Virus genes

Oligo Name	nt	Transcript Length (bp)	Distance from 3' (bp)	Tm (°C)	GC (%)	Design	Sense Probe Sequence (5' to 3')
EBNA1_AD.1	60	1131	202	76.1	45.0	AD	TTTGAAGGATGCGATTAAGGACCTTGTTATGACAAAGCCCGCTCCTACCTGCAATATCAG
EBNA1_AD.2	60	1131	803	79.0	55.0	AD	GAGAGGTCGTGGACGTGGAGAAAAGAGGCCCAGGAGTCCCAGTAGTCAGTCATCATCATC
EBNA2_AD.1	60	1464	687	79.0	53.3	AD	GCACCCTCTTACTCATCAAAGCACCCCAAATGATCCAGATAGTCCAGAACCACGGTCCCC
EBNA3A_AD.1	60	1360	29	76.9	48.3	AD	GAAGCCATTCTCCGCAGGTTTCCACTAGATCTAAGAACACTTCTTCAAGCGATTGGAGCC
EBNA3C_AD.1	60	2973	129	79.2	53.3	AD	GACCTGCCCGGTGTTCCCAAGCTACTGCTGAAGCACAGGAGATTCTCAGTGACAATTCTG
EBNA3C_AD.2	60	2973	893	79.1	51.7	AD	TAAGGCCCAGCCCATAGAAAGTTCACACTTGAGTTCCATGTCGCCCACACAGCCGATATC
LMP1.70 mer	70	2038	813	88.3	47.1	PE	CTGTTCATCTTTGGCTGCTTACTTGTCTTCGGTATCTGGATCTACTTCTTGGAGATTCTCTGGCGGCTTG
LMP 2A&2B.70 mer	70	1196	53	94.5	54.3	PE	CCGACCCCATATCGCAACACTGTATAAAGAATGCCCACCAGATCGCCTGCCACTTCCACAGCAATGGCAC
LMP2_AD.1	60	1196	199	77.0	48.3	AD	AATGGCGACCGTCACTCGGACTATCAACCACTAGGAACCCAAGATCAAAGTCTGTACTTG
BZLF1.70 mer	70	1767	280	90.3	47.1	PE	ACGCACACGGAAACCACAACAGCCAGAATCGCTGGAGGAATGCGATTCTGAACTAGAAATAAAGCGATAC
gp85.70 mer	70	2121	90	93.3	51.4	PE	TCCACGTTCACTATCTGCTGCTGACCACCAACGGGACTGTCATGGAAATTGCGGGCCTGTATGAAGAAAGAG
gp85_AD.1	60	2121	491	76.8	48.3	AD	ATTATCCCGCTCATCAATGTGACATTCATAATCTCTAGTGACCGTGAGGTCCGAGGCTCG
gL_AD.1	60	414	294	77.7	48.3	AD	CGCGTTGGAAAACATTAGCGACATTTACCTGGTGAGCAATCAGACATGCGACGGCTTTAG
g42_AD.1	60	672	259	76.0	46.7	AD	CAACGCCCGATATTCTACCTGTGGTAACTAGAAATCTGAATGCCATTGAGTCCCTTTGGG

AD: ArrayDesign; PE: Primer Express

### Comparison of quantitative EBV gene transcription profiling in a panel of BL cell lines

**Figure 2:**
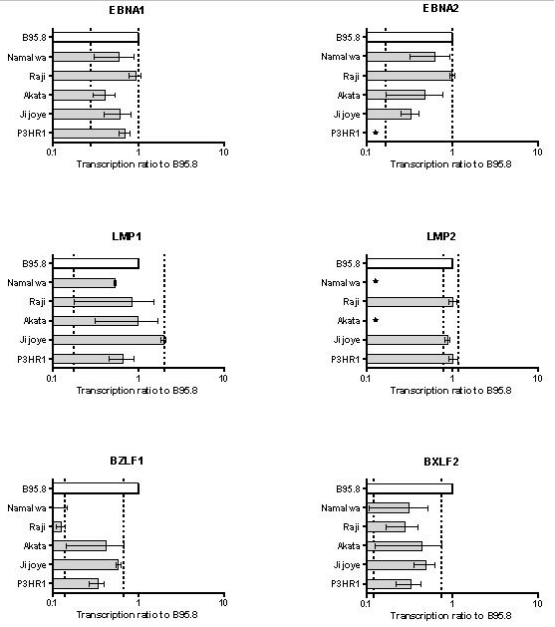
**Quantitative analysis of EBV gene transcription in cultured BL cell lines at steady state**. EBV gene transcription levels in exponentially growing cultured cells were determined by competitive hybridization to the reference cell line B95.8. Shown are mean ± SD values of dye-swap microarray experiments expressed as transcription ratio to B95.8. Dotted lines indicate the range of mean transcription values. Stars represent "not detected."

We next sought to compare quantitative EBV gene transcription profiles in a panel of EBV-harboring BL cell lines. The reference cell line B95.8 displays a latency III expression pattern, but about 5% of the cells display lytic EBV infection. Thus, B95.8 is expected to transcribe both lytic and latent genes. By selecting a set of housekeeping genes that show the same specificity for human and marmoset B-cell lines, we could use B95.8 RNA as a reference in competitive hybridization experiments. RNAs extracted from the EBV-positive BL cell lines Namalwa, Raji, Akata, Jijoye, and P3HR1 were labeled and competitively hybridized against B95.8 labeled RNA in dye-swap experiments (Cy3–Cy5) (Fig. 2).

We found that EBV gene transcription in all BL cell lines tested was, in general, lower than in B95.8, as expected (indicated by fold transcription levels equal or smaller than 1 in Fig. 2). Consistent with our hypothesis, *EBNA1 *mean transcription levels were quite similar in the BL cell lines: their transcription ratios to B95.8 ranged from 0.4 to 0.9 (a 2.25-fold difference), regardless of expected latency I or switch to latency III, or episomal or integrated status of EBV. Transcription levels of *EBNA2 *were highest in Raji, reaching levels observed in B95.8. In Akata cells, transcription levels of *EBNA2 *showed low absolute values, resulting in a greater SD than in the other cells lines. This is in agreement with a latency I pattern, expected for Akata. In P3HR1 cells, *EBNA2 *transcription was below detection levels, as expected from the partial deletion of the *EBNA2 *gene in the genomic EBV sequence present in P3HR1. Mean transcription levels of *EBNA2 *in the other BL cell lines ranged between 0.33 and 0.98 (a fourfold difference). Transcription levels of *LMP1 *among the BL cell lines tested were highest in Jijoye, reaching levels twofold higher than B95.8. *LMP1 *mean transcription ratios to B95.8 in the BL cell lines ranged between 0.53 and 1.9 (a 3.6-fold difference). Absolute transcription levels for Akata were very close to the detection limit (data not shown), resulting in large SDs in the ratios to B95.8. Thus, the results for *LMP1 *transcription in Akata should be considered as being negative. *LMP2 *transcription was not significant in Namalwa and Akata cells. The levels for Raji, Jijoye and P3HR1 were the same as in B95.8. Notably, transcription values for B95.8 were close to saturation, and therefore, the ratios appear especially compressed for *LMP2*.

As expected, EBV lytic gene transcription was lower in the selected BL cell lines than in B95.8. *BZLF1 *transcription ratios varied between 0.1 and 0.6 (a sixfold range) and were very low in all BL cell lines. In Namalwa and Raji cells, transcription was 10% of that of B95.8 (i.e., at the detection limit of microarray). Absolute transcription values of *BZLF1 *were lowest in Akata cells (not shown), resulting in large SD in the ratios to B95.8. Similarly, transcription levels of *BXLF2 *were significantly lower in all BL cell lines than in B95.8, with ratios ranging form 0.3 to 0.5. The absolute values were close to the detection limit for all cell lines (also for B95.8), resulting in large SD values, and the results must be considered essentially negative.

Thus, the BL cell lines exhibited no large differences in their levels of *EBNA1 *gene transcription, regardless of latency patterns that can switch from latency I to latency III in vitro or integration status of the EBV genome. Transcription levels of lytic EBV genes in BL cell lines were lower than in B95.8, but among the BL cell lines tested, transcription was high in producer cell lines (permissive) such as Jijoye and P3HR1 and low (up to 10-fold) in Namalwa (integrated EBV) and Raji (non-producer).

### Validation of EBV microarray results by quantitative real-time PCR

To validate the microarray results obtained by competitive hybridization against the B95.8 cell line, RNAs extracted from the EBV-positive BL cell lines Namalwa, Raji, Akata, Jijoye, and P3HR1 were reverse-transcribed to cDNA, and EBV gene transcription was measured with specific quantitative real-time PCR (qPCR) primers and probe systems. The transcription values were normalized to the transcription levels of *HMBS*, one of the normalization housekeeping genes selected by microarray. *HMBS *was chosen instead of *ACTB *because the transcription values (C_T_: cycle threshold that quantifies the presence of target) of the HMBS assay were closer to the values observed with the EBV-specific qPCR assays (not shown) and therefore should allow more accurate normalization.

**Figure 3:**
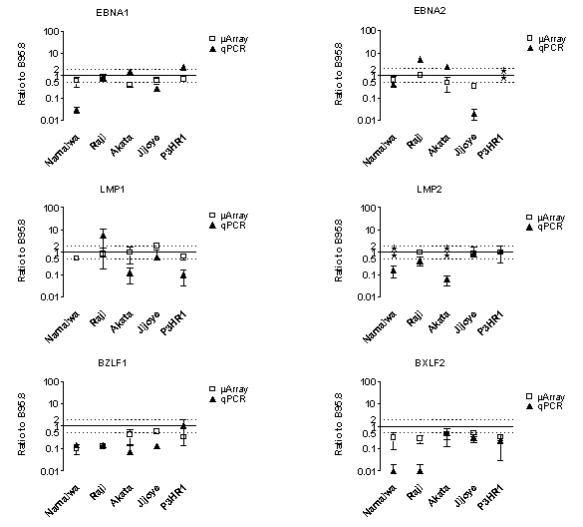
**Validation of microarray results by qPCR analysis**. EBV gene transcription levels in exponentially growing cultured cells were determined by competitive hybridization to the reference cell line B95.8 and by qPCR. Shown are mean ± SD values from dye-swap microarray experiments (open squares) and for three independent qPCR experiments normalized over B95.8 (closed triangles). Stars represent "not detected."

Transcription data from the dedicated microarray were compared to transcription data obtained from qPCR. To allow this comparison, qPCR data, which were normalized to *HMBS *transcription, were transformed in transcription ratio to B95.8 values (Fig. 3). Results from microarray and qPCR were in good agreement when scoring the EBV gene transcription levels as higher or as lower than that in B95.8. However, some discrepancies were observed in the absolute transcription differences. Results from qPCR confirmed that transcription levels of *EBNA1 *do not significantly differ among the BL cell lines, except in Namalwa. In Namalwa, transcription levels were 97% lower from qPCR and about 50% lower measured by microarray. The reasons for this discrepancy are not clear, but they might be due to a polymorphism in the *EBNA1 *gene in Namalwa or, although transcription levels of *HMBS *seemed constant, to the different normalization procedures. Quantitative PCR confirmed microarray results for *EBNA2*, except for Raji, in which transcription levels were a 3.8-fold higher than in B95.8 by qPCR, and similar to B95.8 by microarray.

Transcription levels of *LMP1 *showed the greatest discrepancies between microarray and qPCR. Namalwa and Jijoye were both confirmed by qPCR to transcribe *LMP1 *at the same levels as B95.8. Transcription levels of *LMP1 *in Akata and P3HR1 obtained by qPCR were only 10% of those from the microarray, where the absolute transcription levels for Akata were considered negative. In Raji, transcription levels of *LMP1 *measured by qPCR were fivefold higher than by microarray. The transcription levels of *LMP2 *in Namalwa and Akata cell lines, undetectable by microarray, were confirmed by qPCR, which detected *LMP2* at 10- to 20-fold lower levels, respectively. *LMP2 *transcription levels for Raji, Jijoye, and P3HR1 were similar to those for B95.8 by qPCR, again confirming the microarray data. *BZLF1 *transcription was not detected in Namalwa nor Raji cells and was detected at very low levels in Akata, confirming microarray observations. Levels of *BZLF1 *transcription measured by qPCR were lower (sevenfold) than measured by microarray (1.7-fold).*BXLF2 *transcription in Akata, Jijoye, and P3HR1 was confirmed by qPCR to be twofold to fourfold lower than in B95.8. *BXLF2 *transcription was not detected in Namalwa nor Raji, indicating that the low ratios observed by microarray are actually negative transcription values.

Thus, the qPCR results generally validated the microarray results that transcription levels of *EBNA1 *did not significantly differ among BL cell lines. The qPCR also confirmed the gene transcription patterns that indicate a switch to latency III or permissiveness for lytic EBV infection. Importantly, these results show that the dedicated EBV ODN chip is useful for quantifying latent and lytic EBV gene transcription.

### EBV gene transcription profiling upon induction of lytic infection in Akata cells

**Figure 4:**
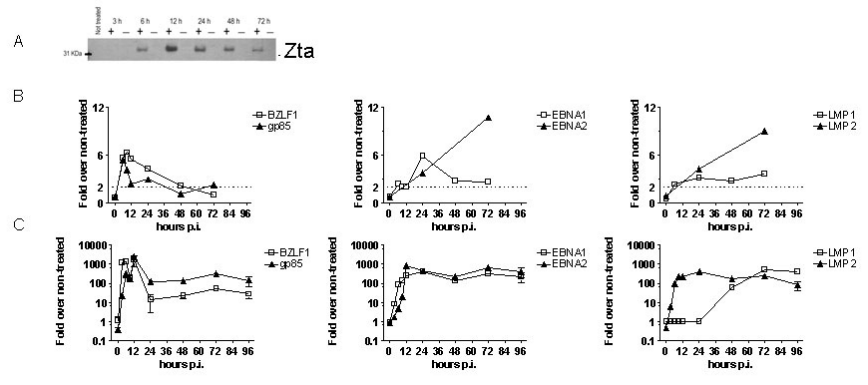
**Effect of induction of lytic EBV infection on Zta and EBV gene transcription**. (A) Western blot showing protein transcription levels of Zta in IgG cross-linking induced (+) and non-treated (-) Akata cells. (B) Transcription of EBV genes was quantified by microarray analysis in Akata cells upon induction of lytic infection. Treated and non-treated cells were harvested at different times after induction of lytic infection by BCR cross-linking. Competitive hybridization of labeled samples from treated cells was performed against non-treated cells. Shown are mean values of two dye-swap experiments. (C) EBV gene transcription was quantified during induction of lytic EBV infection in Akata cells by BCR cross-linking. For each time point, treated and non-treated cells were harvested and subjected to qPCR. Each point represents the difference between induced and non-induced cells, normalized to the HMBS housekeeping gene. Results are from at least two biological replicates and are given as: ΔΔC_T _= (C_T_(EBV gene)-C_T_(HMBS)}_treated_-{C_T_(EBV gene)-C_T_(HMBS))_not treated._

Finally, we wondered whether the chip would allow us to record quantitative changes in EBV gene transcription upon a treatment intervention. Lytic infection can be efficiently induced by IgG cross-linking of the B-cell receptor in Akata cells [17]. The key events are activation of the master lytic EBV gene *BZLF1 *and expression of its product Zta [15]. Induction of lytic infection was confirmed by detecting Zta protein expression by western blotting (Fig. 4A). As expected, Akata cells were negative for Zta before induction, and the maximal expression level of Zta was observed at 12 h after induction.

We then analyzed the simultaneous transcription of EBV genes with the dedicated EBV microarray chip (Fig. 4B). To quantify EBV gene transcription, RNA from treated cells was competitively hybridized against RNA from non-treated cells collected at the same time, with dye-swap. Twofold or higher differences in transcription were arbitrarily considered significant changes when the standard deviation was not above twofold. *BZLF1 *and *BXLF2*/gp85 were induced more than fivefold at 6 h, and their transcription declined 48 h after treatment. Similarly, transcription of *BKRF2*/gp42 and *BZLF2*/gL increased at 6 h, peaked at 24 h, and declined at 72 h (not shown). The latent genes, including *EBNA2*, *LMP2 *and *EBNA3A*, *EBNA3C *(not shown), were up-regulated more than threefold 24 h after stimulation. Transcription of the latent genes *EBNA1 *and *LMP1 *was up-regulated twofold 6 h after induction in Akata and persisted for 72 h with a peak at 24 h. Akata cells unexpectedly exhibited a significant increase in transcription levels of the latent EBV genes. The increase of transcription of *BZLF1 *peaked at sixfold over non-induced cells at 12 h and was terminated when transcription of *EBNA1*, *EBNA2 *and *LMP2 *increased at 24 h after induction.

qPCR obtained the results from the microarray for transcription profiles of EBV genes after B-cell receptor cross-linking in Akata cells (Fig. 4C). Transcription of *BZLF1 *and *BXLF2*/gp85 was observed with qPCR also 6–12 h after induction, followed by increases in levels of *EBNA1*, *EBNA2*, and *LMP2 *gene transcription.*LMP1 *transcription was also detected by qPCR at a significant level at 48 h after induction, later than the increase of *EBNA1*, *EBNA2*, and *LMP2 *24 h after induction. The increase in gene transcription levels observed by qPCR was much larger (over 100-fold) than by microarray (about 10-fold).

In summary, transcription of lytic EBV genes and a slightly deferred increase in transcription of latent genes upon induction of lytic infection in Akata cells could be quantitatively determined. This finding suggests that the dedicated EBV chip is suited for quantitative analysis of simultaneous EBV gene transcription also after interventions leading to alteration of gene expression.

## Discussion

In this work, we report a novel assay system that quantitatively and simultaneously determines levels of transcription of EBV genes. This system will contribute to an improved understanding of EBV gene transcription regulation and its impact on B-cell biology. Specifically, we showed that (i) quantitative transcription of housekeeping genes considerably differs between BL cell lines and that selection of housekeeping genes appropriate for normalization is an essential prerequisite to allow for comparison of quantitative EBV gene transcription between cell lines; (ii) the transcription levels of *EBNA1 *in BL cell lines do not significantly differ in contrast to transcription levels of other EBV genes; and (iii) the dedicated EBV chip is sensitive enough to detect EBV gene transcription changes triggered by treatment interventions. Our results suggest that *EBNA1 *transcription levels are uniform in BL and that microarray analysis can reveal refined insights on housekeeping and EBV gene transcription behavior.

### A BL-specific housekeeping gene set

The importance of the choice of genes for data normalization has become increasingly evident with the advent of high-throughput gene-profiling technologies, such as microarray and qPCR. A comprehensive literature analysis of expression studies published in high-impact journals during 1999 indicated that *GAPDH*, *ACTB*, 18S and 28S rRNA were used as single control genes for normalization in more than 90% of cases [12]. Because expression of these genes can vary considerably [9,10], the validity of the conclusions depends heavily on appropriate controls.

We defined a set of eight housekeeping genes with similar transcription levels in the five BL cell lines tested and, most importantly, in the marmoset LCL B95.8, the reference cell line for EBV. Seventeen housekeeping genes from a compendium of 451 housekeeping genes expressed in most tissues were tested them on a panel of BL cell lines. Genes with the most or most constant expression and for which a probe sequence was available in the compendium for open-source human probes were selected [11]. Most remarkably, the most highly expressed genes could not be detected in all cell lines or gave the inconsistent results. Fortunately, the most consistently expressed genes proved to be much better, with a coefficient of variation lower than 0.6. The best housekeeping gene in our set was *ACTB*, which had the smallest coefficient of variation of all genes.

Absolute quantification of gene transcription by microarray is accurate when enough probes are used to allow global normalization (typically several thousands) or when a reference is used to normalize results. On a microarray containing fewer than 1,000 elements, measurements tend to be more variable than those from qPCR. Nevertheless, the general overlap between microarray data normalized over the set of housekeeping genes we selected and qPCR data normalized over *HMBS *transcription levels (part of the selected housekeeping set) indicate that the housekeeping gene improves the accuracy of results. Thus, the qualitative transcription profile obtained with the housekeeping gene normalization set was very close to that obtained by qPCR. Importantly, this set of housekeeping genes will be useful in other microarray experiments since the cell lines are widely used to study immunoglobulin rearrangements and other cellular processes, such as DNA repair and apoptosis [18-22].

### Validation of the dedicated EBV chip: advantages and disadvantages over qPCR

The dedicated EBV chip was validated by comparing microarray and qPCR results. In general, the two techniques agreed in distinguishing genes transcribed or not transcribed, but some substantial differences occurred in the quantitative assessment of gene transcription. Several reasons might account for differences. First, the systems do not target identical gene sequences, and differences in the efficiency of reverse-transcription of mRNAs might occur, even though we used the most homogeneous design possible for all probes. Second, differences in the amplitude of linear detection for microarray and qPCR (2 log_10 _vs 4 log_10_) might explain why microarray transcription levels are more "compressed" than qPCR transcription levels (i.e., microarray levels range from 0.1 to 10; qPCR values range from 0.01 to 100).

The use of long ODN-based microarrays has two major advantages over qPCR. The chip can be quickly expanded to cover additional cellular genes, thereby decreasing the cost per probe considerably. A microarray ODN chip can easily accommodate up to 42,000 probes [23]. Another advantage is the lower sensitivity to single nucleotide polymorphisms. The lower sensitivity could be considered a disadvantage, but in the case of viruses which show a high degree of polymorphism, it is an advantage. In fact, the lower sensitivity helped in the present work. The *BZLF1*-specific qPCR assay was designed using the B95.8 sequence, and one of the primers failed to work in Akata cells because of a polymorphism at the 3' end of the primer sequence (not shown). By contrast, the microarray did not show a dramatic effect on the detection efficiency between B95.8 and Akata, although the same polymorphism is within the sequence of the probe BZLF1.70 mer. Therefore, microarray probes are more flexible and less prone to false negative results than qPCR systems and thus more suitable for transcription profiling of patient samples where multiple, not fully sequenced, EBV strains may be present [24]. In conclusion, an EBV ODN microarray is a valid alternative to qPCR and other techniques, especially for analysis of quantitative transcription of a large number of genes or of patient samples, where the exact sequence of the EBV strain is not known.

#### Transcription levels of *EBNA1 *do not differ significantly among BL cell lines

Qualitative expression of EBV genes has been extensively studied, but our results are the first quantitative analysis. The BL cell lines were selected to cover as many as characteristics of EBV infection as possible. Notably, transcription levels of *EBNA1 *are quite constant across BL cell lines, despite differences in EBV genome integration status, EBV type, EBV latency pattern or permissivity. This observation suggests tight transcriptional control of *EBNA1*, the gene mainly required for maintenance of the episomal EBV genomes. Since Namalwa cells carry two copies of EBV integrated in their genome and are expected to display latency I expression pattern (only EBNA1), transcription of *EBNA1 *might not be required for EBV maintenance. The transcription levels, similar to those in the BL cell lines with episomal EBV, could indicate that *EBNA1 *transcription is needed for other functions, such as cell proliferation. The low transcription levels of *EBNA2 *and *LMP*s and the absence of lytic gene transcription in Namalwa confirm its tight latency pattern I expression. In Raji cells, also expected to express latency I, we observed a switch to latency III, with transcription of *EBNA2 *and *LMP1 *at the same levels observed in B95.8. Of course, due to the limited number of BL cell lines investigated, no conclusions can be drawn. Nevertheless, the validated microarray will make it possible to further investigate transcription levels of EBV genes, their correlation with cellular gene transcription, and the mechanisms regulating translation of EBV genes into proteins, all crucial steps allowing EBV to hide from the immune system and persist for life in host B-cells.

### Transcription levels of latent EBV genes increase upon induction of lytic infection

Induction of lytic infection by B-cell receptor cross-linking of Akata cells resulted in increased transcription levels of latent EBV genes, which peaked concomitantly with termination of increased *BZLF1 *gene transcription and protein expression. This result comes partially as a surprise: the extent and kinetic of increase of latent EBV gene transcription have not been described in a quantitative manner. Previous work had shown that translation of *EBNA1*, *EBNA2*, and *LMP1 *increased after induction of lytic infection [25]. An increase in *LMP2 *expression was also observed in Akata cells by Southern reverse transcription PCR [25], but our analysis of the kinetics of latent EBV genes transcription indicates that their transcription is increased concomitantly with early lytic EBV genes. EBNA2 can induce lytic infection in Akata cells under some circumstances [26], but LMP1 [27] and LMP2 [28] exert a negative effect on induction of lytic infection. From the microarray data, one might hypothesize a novel mechanism in which transcription of latent genes regulates activation of lytic infection.

## Conclusion

Using a newly developed dedicated EBV microarray ODN chip containing a set of carefully selected housekeeping genes for data normalization, we defined the quantitative profile of EBV gene transcription of a panel of BL cell lines. Furthermore, we showed that *EBNA1 *transcription levels are similar across BL cell lines, suggesting tight transcriptional control of *EBNA1*. Finally, we showed that the dedicated EBV chip can be used to monitor quantitative latent and lytic EBV gene transcription after induction of lytic EBV infection. The ability to quantify EBV gene transcription will allow studies of EBV gene translation. This result is particularly important for considering EBV as a target for therapies of EBV-positive tumors. The selected housekeeping gene sequences and EBV-specific sequences can be easily incorporated in other dedicated microarrays and will be useful for studies of cellular and EBV gene transcription profiles.

## Methods

### Cell lines

As a reference for EBV gene transcription, we used the EBV-positive B95.8 cell line, an LCL of marmoset origin that expresses latent and lytic EBV genes both constitutively and concomitantly. The EBV-negative BL cell line BJAB served as negative control. To characterize quantitative EBV gene transcription, we chose a panel of EBV-harboring BL cell lines with different characteristics. The panel included three BL cell lines with EBV type I virus: Namalwa [29], Raji [30] (50 copies of EBV per cell [31] with a deletion in EBNA3-C [32], and Akata [26] (20 copies of EBV per cell). It also contained two BL cell lines with EBV type II virus: Jijoye and its daughter cell line P3HR1, which has a deletion in EBNA2. Cells infected with EBV type I and type II exhibit different transformation and outgrowth potentials [33]. Jijoye and P3HR1 are EBV-producing cell lines, Akata cells can be induced to produce EBV, and Raji and Namalwa cells do not produce EBV particles. Furthermore, in Namalwa cells, the EBV genome is integrated in the human chromosome [34], while the other BL cell lines harbor the virus in episomal form. Finally, although BL cells in culture initially display an EBV latency I pattern as BL cells do *ex vivo*, some BL cell lines (e.g., Raji) may switch to a latency III pattern upon continued in vitro culture, and others (e.g., Namalwa) do not. Akata was a kind gift from Dr. A. Bell (Birmingham, UK); all other cell lines were from ATCC (Rockville, MD). Cells were cultivated in RPMI 1640 with 10% fetal bovine serum at 37°C and in 5% CO_2 _humidified atmosphere.

### Induction of lytic EBV infection

Lytic EBV infection was induced by cross-linking of surface immunoglobulin with anti-IgG as follows. For 3 h, 10^6 ^Akata cells were incubated with 100 μg/ml anti-human IgG (Dako A0423, DakoCytomation, Zug, Switzerland), and after a medium change, the cells were plated on 24-well plates [7]. Cells were harvested before and at different times after stimulation and subjected to total RNA extraction or western blotting. Control cells were handled in the same way as the test cells but not incubated with anti-human IgG.

### Isolation, amplification, and labeling of nucleic acids

For experiments that induced lytic EBV infections, total RNA was isolated from cells with RNeasy midi kit or mini kit (Qiagen, Basel, Switzerland), according to the manufacturer's instructions. Limiting amounts of total RNA (1 μg) were amplified and then labeled with the amino allyl MessageAmp aRNA Kit (Ambion Europe, Huntingdon, UK). Indirect amino allyl labeling was performed with CyScribe Post-Labeling Kit (Amersham Bioscience, Dübendorf, Switzerland). Cy-dyes incorporation was measured with Nanodrop-1000 (NanoDrop Technologies, Wilmington, DE, USA). The validity of the amplification protocol was tested by competitive hybridization of cRNA from the reference cell line B95.8 labeled with Cy3 vs B95.8 cRNA labeled with Cy5. Both labeled cRNAs were obtained with the direct labeling protocol (non-amplified). A significant correlation factor was observed between amplified and non-amplified samples (R = 0.95).

### Design of probes and array fabrication

Sequences for some housekeeping genes were derived from the Church's published set of array probes [11]. Probes were designed either using the Primer Express software (PE, Applied Biosystems, Rotkreuz, Switzerland), with parameters: 40–60% GC content, T_m _70–90°C, and lack of homo-oligomers and sequence repeats (labeled gene-name.70 mer), or the ArrayDesigner 2 (AD, Premiere Biosoft International, Palo Alto, CA, USA) with parameters: 60–70 nucleotides (nt), distance from 3,500 nt or 1,000 nt (labeled gene-name_AD). Probes were designed to be as close to the 3' end as possible, distances from 3' ranging from 20 nt to 1500 nt (median 407 nt). Probes T_m _ranged between 75°C and 95°C (median 77.7°C); GC content ranged from 40% to 60% (median 51.6%). (For details, see Table 1.) 5' Amino C6 PAGE-purified probes were synthesized by Sigma-Genosys (Pampisford, UK). Slides coated with 3-glycidoxypropyl-trimethyl silane ("epoxysilane") were obtained from Scienion (Berlin, Germany). 20 μM solutions of ODN in 3 × SSC were spotted using a Biochip Arrayer later a Piezorray (both from PerkinElmer Life Sciences, Seer Green, UK). 3 droplets (about 1 nL) were deposited at the slide surface. ODNs were immobilized in a humidity chamber (23°C, 75% relative humidity) over night.

### Microarray hybridizations

Hybridizations were performed in an automated hybridization station (HS4800, Tecan, Salzburg, Austria) for 16 h at 42°C. Slides were washed with 2 × SSC/0.2% SDS, 0.2 × SSC/0.2% SDS, finally 0.2 × SSC, and dried in the machine with a flow of nitrogen.

### Microarray experiments and analysis of data

The reproducibility of microarray results was tested by performing a set of experiments in which RNA isolated from the same cell line was labeled with either Cy3 or Cy5 and then competitively hybridized (self vs self). The correlation coefficient (R) of self vs self hybridization was 0.96–0.99. Slides were scanned at decreasing laser power (70, 60, 50, 40, and 30 db) with an Affymetrix 427 laser scanner (MWG Biotech, Ebersberg, Germany). Spot intensities from TIFF (Tagged Image File Format) files were analyzed with ImaGene 5.0 software and imported in GeneSight 3.2 software (BioDiscovery, El Segundo, CA, USA). Local backgrounds of spot intensities were averaged and subtracted (median). Cy3 and Cy5 intensities were corrected by normalization (division by the median of the housekeeping genes). Replicate spots were processed singularly and combined after ratios were calculated (mean ± SEM). Ratios between competitive hybridizations represent the mean value of dye-swap experiments. When more than one probe per gene was used, the mean value (± SEM) of all probes was calculated.

### Quantitative real-time PCR assays of EBV gene transcription

For qPCR assays of EBV gene transcription analysis, 1 μg of total RNA was reverse transcribed with oligo-dT (15) and Omniscript reverse transcriptase (Qiagen, Basel, Switzerland). The design and validation of the primers and probes specific for EBV were as described (Berger C, Bonanomi A, Ladell K, Nadal D. submitted for publication). Probes were labeled with 5'6-carboxyfuorescein (FAM) and 3' Black Hole Quencher (BHQ, Biosearch Technologies, Novato, CA, USA). qPCR was performed in a reaction volume of 15 μl with the ABI-TaqMan Master Mix with uracil-N-glycosylase (Applied Biosystems, Rotkreuz, Switzerland) [35]. The normalized transcription values correspond to 2^-C^_T_^(EBV)^^-C^_T_^(HMBS) ^= 2^-ΔC^_T_, where C_T _is the cycle threshold number that quantifies the target present.

### Western blotting

For western blotting, cells were lysed using a syringe in lysis buffer (125 mM Tris-HCl, pH 6.8, 6% SDS). The proteins were separated by electrophoresis on a 10% Bis-Tris gel (Invitrogen, Basel, Switzerland). For immunoblotting, the following antibodies and dilutions were used: anti-EBV Z Replication Activator (ZEBRA, Zta), clone AZ-69 (1:100 dilution, Argene Biosoft, Varilhes, France), the monoclonal rat-anti LMP2A, (clone TP 14B7, 1:100 dilution, Ascenion, Germany), the mouse anti EBNA2 (DakoCytomation, Baar, Switzerland), and the PCNA (BD Bioscience, Basel, Switzerland). Antibodies were visualized with ECL Western Blotting Detection Reagent (Amersham Bioscience, Dübendorf, Switzerland).

## Abbreviations

AD, ArrayDesign software; BL, Burkitt's lymphoma; C_T_, cycle threshold; CV, coefficient of variation; EBV, Epstein-Barr virus; LCL, lymphoblastoid cell line; LMP, latent membrane protein; ODN, oligonucleotide; PCR, polymerase chain reaction; PE, Primer Express software; qPCR, quantitative real-time polymerase chain reaction; SD, standard deviation.

## Competing interests

The author(s) declare that they have no competing interests.

## Authors' contributions

MB, CB, FKN, and DN conceived the study and designed the experiments. MB, CB, JAS, JS, and AB participated in the experimental data collection. MB analyzed the data. MB, CB, and DN drafted the manuscript.
